# Associations Between Added Sugars Intake from Various Food and Beverage Sources and Diet Quality Among the U.S. Population

**DOI:** 10.3390/nu16244333

**Published:** 2024-12-16

**Authors:** Laurie Ricciuto, Loretta DiFrancesco, P. Courtney Gaine, Maria O. Scott, Victor L. Fulgoni

**Affiliations:** 1 Department of Nutritional Sciences, University of Toronto, Toronto, ON M5S 1A8, Canada; 2 Source! Nutrition, Toronto, ON M6S 5A6, Canada; 3 The Sugar Association, Inc., Washington, DC 20005, USA; gaine@sugar.org (P.C.G.); mscott@sugar.org (M.O.S.); 4 Nutrition Impact, LLC, Battle Creek, MI 49014, USA; vic3rd@aol.com

**Keywords:** added sugars, diet quality, Healthy Eating Index, NHANES, U.S. population

## Abstract

**Background:** A diet high in added sugars has been linked to poor diet quality; however, little is known about specific sources of added sugars and their association with diet quality. **Objective:** This study examined associations between added sugars intake from specific food and beverage sources and diet quality, as indicated by the Healthy Eating Index (HEI) 2020 score, among the U.S. population. **Methods:** Data from eight consecutive 2-year cycles of the National Health and Nutrition Examination Survey (2003–2004 through 2017–2018) were pooled, and regression analysis was conducted to examine associations between total HEI-2020 score or HEI-2020 component scores and added sugars intake (% kcal) from key contributors: soft drinks, fruit drinks and coffee and tea; ready-to-eat cereals; flavored milk; sweet bakery products; and snack/meal bars. **Results:** A higher added sugars intake from soft drinks, fruit drinks and coffee and tea was associated with lower diet quality (lower total HEI score and lower scores on most of the HEI components) among both children and adults (*p* < 0.0001). In contrast, higher added sugars intakes from flavored milk (*p* < 0.0001) and snack/meals bars (*p* < 0.001) among children, and from sweet bakery products (*p* < 0.0001) among adults, were associated with higher diet quality. For all these associations, changes in the total HEI score across quintiles of added sugars intake were very small, ranging from 50.2 to 52.8 for children and 55.4 to 57.5 for adults, depending on the added sugars source. **Conclusions:** The nature of the relationship between added sugars intake and diet quality depends on the source of added sugars. While the small differences in diet quality may be of limited practical significance, our results suggest that the consideration of the different roles of various added sugars sources in the diet is warranted when developing dietary guidance.

## 1. Introduction

National dietary guidelines provide recommendations for healthy eating and are the basis of nutrition programs and policies aimed at meeting the nutritional needs of a population for maintaining good health and reducing the risk of chronic disease. Key elements of a healthy eating pattern recommended in the 2020–2025 Dietary Guidelines for Americans (DGA) include adequate amounts of fruits and vegetables, greens and beans, whole grains, dairy, protein foods (particularly seafood and plant protein) and fatty acids, and limited amounts of refined grains, sodium, added sugars and saturated fats [1]. These dietary elements are all components of the Healthy Eating Index (HEI), which is a scoring tool developed by the U.S. Department of Agriculture (USDA) designed to assess how well diets align with recommendations in the DGA; a higher HEI score denotes a better quality diet [2]. The HEI has been updated since 2005 in concordance with updates to dietary recommendations and is widely used in research examining diet quality; the most current HEI-2020 reflects the 2020–2025 DGA [3].

Examinations of added sugars intake and diet quality have used a variety of indicators to define diet quality, including food choices, nutrient intakes and indices, such as the HEI. A systematic review has demonstrated that an increased intake of added sugars (g or % kcal) was consistently associated with poorer diet quality (lower diet index score, lower intake of nutritious foods or higher intake of discretionary foods) across a wide range of populations age ≥ 2 y; however, the effect sizes were small to moderate [4]. Individual studies on the U.S. population ≥ 2 y have shown increases in the prevalence of inadequacy of several micronutrients across deciles of added sugars intake, and they have identified a threshold of intake (18% kcal) above which those associations were significant [5,6]. Added sugars intake from sweetened beverages (e.g., soft drinks, fruit drinks) are a particular concern, and studies have shown lower total HEI scores among consumers of sweetened beverages compared to non-consumers, typically on the order of 6 to 11-point differences, depending on the age group [7,8,9]. However, when specific sweetened beverages have been examined individually, associations with HEI score varied depending on beverage type [7,10], suggesting associations between added sugars intake and diet quality might vary by the source of added sugars.

Although sweetened beverages are key contributors to added sugars intake, food sources of added sugars also make significant contributions, with one study estimating the top food sources together account for 31–51% of total daily added sugars intake among the U.S. population [11]. Studies on associations between added sugars from foods and diet quality are scarce. One study among Canadian children has shown lower total HEI scores across tertiles of added sugars intake from foods (mean HEI score of 79 down to 73) [12]. Other studies have been limited to examining how diet quality differs between consumers and non-consumers of specific foods, such as ready-to-eat cereals (RTEC) [13], or how diet quality varies among different snacking patterns [14,15], which may be correlated with added sugars intake.

There is a paucity of research examining how the association between added sugars intake and diet quality might vary among different food and beverage sources of added sugars. Recommendations to reduce added sugars in the diet capture all sources of added sugars, but some sources belong to food groups whose consumption is encouraged, such as dairy and whole grains; thus, programs or policies restricting foods in these groups would contradict messaging on overall diet quality. Understanding potential differences in the relationship between added sugars intake from various food and beverage sources and diet quality can help to inform dietary recommendations regarding added sugars intake.

The purpose of this study was to examine associations between added sugars intake from specific food and beverage sources and diet quality, as indicated by the HEI-2020 score, among the U.S. population. The following sources of added sugars were selected for analysis based on their contribution to added sugars intake and/or their role in the overall diet, namely: a select beverage group (soft drinks, fruit drinks and coffee and tea); RTEC; flavored milk; sweet bakery products; and snack/meal bars.

## 2. Methods

### 2.1. Data Sources

Information on the diet and health of the U.S. population is collected on a regular basis through NHANES, which is a nationally representative cross-sectional survey of non-institutionalized civilian residents conducted by the U.S. National Center for Health Statistics, which is part of the U.S. Centers for Disease Control and Prevention. All NHANES participants provide written, informed consent. Details on the NHANES survey design, data collection and analytic procedures are reported elsewhere [16,17].

Data from eight consecutive 2-year cycles of NHANES (from 2003–2004 through 2017–2018) each with two 24 h dietary recalls were pooled to provide sufficient sample sizes to examine individual sources of added sugars. Only subjects with complete dietary intake data from two 24 h recalls as determined by USDA staff were included. Those with zero calories, incomplete records on either of the two days of dietary recall, and those pregnant or lactating were excluded. The final analytic sample size was 21,000 for those 2–18 y and 35,094 for those ≥19 y.

The USDA Food Patterns and Equivalents Database converts foods and beverages reported in the 24 h recalls into food pattern equivalents corresponding to those in the DGA, including added sugars [18]. The added sugars food pattern component comprised caloric sweeteners using the definition of “sugars that are added to foods as an ingredient during preparation, processing or at the table; and do not include naturally occurring sugars, such as lactose present in milk and fructose present in whole or cut fruit and 100% fruit juice” [18].

The USDA, via What We Eat In America (WWEIA), which is the dietary intake component of NHANES, places each food or beverage item reported by study participants into mutually exclusive food categories that group similar foods or beverages based on consumer usage and nutrient content. Each 2-year cycle of NHANES has an associated WWEIA dataset. There are 167 unique food categories in the 2017–2018 data release [19].

### 2.2. Added Sugars Intake and HEI-2020 Scores

In order to compare how various sources of added sugars are associated with diet quality, mean added sugars intake was determined first for context for the following: the total diet (all foods and beverages); all foods (no beverages); and all beverages (no foods). Added sugars intake from specific sources was then determined for a select beverage group comprising soft drinks, fruit drinks (first two digits of WWEIA subgroup 72) and coffee and tea (WWEIA subgroup 73), as the beverages contributing the most to added sugars (soft drinks and fruit drinks) [11] or those whose contribution to added sugars has increased over time (coffee and tea) [20]. Added sugars intake was also determined for other sources of added sugars that have been associated, either positively or negatively, with diet quality: RTEC (first two digits of WWEIA subgroup 46) [13]; flavored milk (first two digits of WWEIA subgroup 12) [21]; sweet bakery products (cakes and pies, cookies and brownies, doughnuts, sweet rolls, pastries) (first two digits of WWEIA subgroup 55) [22]; and snack/meal bars (cereal bars, nutrition bars) (first two digits of WWEIA subgroup 54) [23].

Calculations of added sugars intake (g/day and % kcal) and HEI-2020 scores were based on average intakes across the two days of dietary recall. Quintiles of added sugars intake (Q1–Q5) were determined for the total diet, all foods and all beverages. However, for each of the specific sources of added sugars (the select beverage group, RTEC, flavored milk, sweet bakery products and snack/meal bars), more than 20% of the sample had zero intake (i.e., did not report consuming the source on either of the two days of recall). For these sources, Q1 was set at 0 and comprised all non-consumers, and the remaining sample (those who consumed the source) was divided into four equal intake groups (Q2, Q3, Q4, Q5).

### 2.3. Statistical Analyses

SAS 9.4 (SAS Institute, Cary, NC, USA) software was used for all data management and statistical analyses with primary sampling units, strata and 2-day dietary weights to adjust results for the complex sampling design of NHANES and to provide nationally representative results. Given added sugars is a component of the HEI, an adjusted total HEI score was calculated by summing the non-added sugars components of the HEI and normalizing to 100 points. Analyses were conducted using both actual and adjusted total HEI scores, which yielded similar results, and thus only those for the actual score are presented.

Regression analyses were conducted using total HEI score and HEI component scores as dependent variables with added sugars intake (% kcal) as the independent variable as (1) a continuous variable to assess the linear trend of association of added sugars with total HEI score; and (2) quintiles of intake to assess the trend across distinct levels of added sugars intake and total HEI score (quintile trend). Given the results were generally similar for both analyses, linear trend results are presented, yet we inform whenever quintile trend results were different. Also, given the low number of adult consumers of flavored milk, regression analyses were not conducted for this specific source of added sugars among those ≥19 years old.

## 3. Results

### 3.1. Added Sugars Intake from Food and Beverage Sources

The added sugars intake (g/day and % kcal/day) from all foods and all beverages was similar, though it was slightly higher from all foods compared to all beverages, among both children and adults (Table 1). The select beverage group (soft drinks, fruit drinks and coffee and tea) contributed more than one third of the total added sugars intake among both children and adults (26.4/69.9 = 37.8% and 28.6/68.8 = 41.6%, respectively). For the specific added sugars sources, at least 10% of children and adults reported consuming any one of the sources in the previous 24 h (i.e., consumers), with the exception of flavored milk among adults, with only 4.3% consumers.

Quintile ranges of added sugars intake (% kcal) varied by source, although they were generally similar among children and adults (Table 2). For the select beverage group (soft drinks, fruit drinks and coffee and tea), the top 25% (Q5) of children and adult consumers had daily added sugars intakes above 9.62% and 11.87% kcal, respectively; at the lower end was snack/meal bars with the top 25% (Q5) of both children and adult consumers having daily added sugars intakes above 1.63% and 1.79% kcal, respectively.

### 3.2. Associations Between Added Sugars Intake from Food and Beverage Sources and Total HEI-2020 Score

#### 3.2.1. Children

There was a significant inverse association between added sugars intake from the total diet and total HEI score among children (regression coefficient (Beta) = −0.0978, SE = 0.0057, indicating that for every 1% unit increase in calories from added sugars, the total HEI score decreased 1 point), which could be attributed to the significant inverse association for all beverages (Beta = −0.1477, SE = 0.0070) versus the association for all foods, which was significantly positive (Beta = 0.0511, SE = 0.0094) (Table 3); the inverse association for all beverages was the largest of these three groups. Additionally, there was a significant inverse association between added sugars intake from the select beverage group (soft drinks, fruit drinks and coffee and tea) and total HEI score (Beta = −0.1559, SE = 0.0077), which was slightly larger than the association for all beverages.

For the specific added sugars sources, there were significant and positive associations between the added sugars intake and total HEI score among children for flavored milk (beta = 0.3157, SE = 0.0379) and snack/meal bars (beta = 0.2522, SE = 0.0736) (Table 3). For RTEC and sweet bakery products, the associations were not significant for the linear trend, while they were significant and positive for the quintile trend.

Regardless of these associations, there were only very small changes overall in the total HEI score among children across the quintiles of added sugars intake, ranging from 52.79 to 50.17 for the select beverage group (soft drinks, fruit drinks and coffee and tea); 51.57 to 53.05 for flavored milk; and 51.66 to 52.03 for snack/meal bars (Table 3).

#### 3.2.2. Adults

Similar to children, there was a significant inverse association between added sugars intake from the total diet and total HEI score (beta = −0.0401, SE = 0.0043) among adults, which could be attributed to the significant inverse association for all beverages (beta = −0.0991, SE = 0.0051), given the association for all foods was significantly positive (beta = 0.1426, SE = 0.0076) (Table 4); the positive association for all foods (not all beverages, as in children) was the largest of these three groups. Additionally, there was a significant inverse association between added sugars intake from the select beverage group (soft drinks, fruit drinks and coffee and tea) and total HEI score (beta = −0.0939, SE = 0.0051), which was similar in strength to the association for all beverages.

Compared to children, there were fewer significant associations between the specific sources of added sugars and the total HEI score among adults with only one significant positive association for added sugars intake from sweet bakery products (beta = 0.1519, SE = 0.0140) (Table 4).

Regardless of these associations, similar to children, changes in the total HEI score across the quintiles of added sugars intake among adults were very small, ranging from 57.48 to 55.37 for the select beverage group (soft drinks, fruit drinks and coffee and tea) and from 56.26 to 57.40 for sweet bakery products (Table 4).

### 3.3. Associations Between Added Sugars Intake from Food and Beverage Sources and HEI Component Scores

#### 3.3.1. Children

For most of the sources examined (the total diet, all foods, all beverages, the select beverage group and flavored milk), the associations between added sugars intake and most of the HEI component scores among children were significant and in the same direction as the total HEI score, except for the component scores for total vegetables, total protein foods, fatty acids and saturated fats, which were consistently in the opposite direction (Supplemental Table S1). Similar to the total HEI score, added sugars intake from the total diet, all beverages and the select beverage group was inversely associated with most of the HEI component scores except for the same four component scores (total vegetables, total protein foods, fatty acids and saturated fats), which showed positive associations. Likewise, similar to the total HEI score, added sugars intake from all foods and from flavored milk was positively associated with most of the HEI component scores except for the same four component scores (total vegetables, total protein foods, fatty acids and saturated fats), which showed inverse associations.

#### 3.3.2. Adults

For most of the sources examined (the total diet, all foods, all beverages, the select beverage group and sweet bakery products), the associations between added sugars intake and almost all of the HEI component scores among adults were significant and in the same direction as the total HEI score; the one exception was the saturated fats component score, which was consistently in the opposite direction (unlike among children, where four component scores were in the opposite direction) (Supplemental Table S2). Similar to the total HEI score, added sugars intake from the total diet, all beverages and the select beverage group was inversely associated with most of the HEI component scores except for the saturated fats component score, which showed a positive association. Likewise, similar to the total HEI score, added sugars intake from all foods and from sweet bakery products was positively associated with most of the component scores except for the saturated fats component score, which showed an inverse association.

## 4. Discussion

Our analysis of dietary intake among the U.S. population demonstrated differences in the association between added sugars intake and diet quality depending on the added sugars source. At a broad level, higher added sugars intake from the total diet was associated with lower diet quality, as indicated by lower HEI-2020 total and component scores, which were consistent with other studies [4,5,6,24]. This inverse association between added sugars intake and diet quality was mainly attributable to the added sugars intake from beverages, specifically the combination of soft drinks, fruit drinks and coffee and tea, which is consistent with other studies demonstrating associations between higher added sugars intake from sweetened beverages and lower diet quality [7,8,9,25]. In contrast, our analysis also revealed that higher added sugars intake from all foods was positively associated with higher diet quality with the strongest associations for specific food sources, including flavored milk and snack/meal bars among children, and sweet bakery products among adults.

Differences between children and adults in the nature of the associations that emerged could be indicative of different dietary patterns among these age groups. Among children, added sugars intake from the combination of soft drinks, fruit drinks and coffee and tea was more strongly inversely associated with diet quality than intake from all foods, which was likely due to the prominence of these beverages in their diets, particularly among those 9–18 years old [11,26,27]. In contrast, among adults, the added sugars intake from all foods was more strongly positively associated with diet quality than intake from these beverages, perhaps reflecting a more diverse diet, particularly among older adults, for whom these beverages have a less prominent role in the diet compared to children [11,26]. Furthermore, associations with diet quality emerged for added sugars intake from flavored milk and snack/meal bars among children, and for sweet bakery products among adults, also possibly reflecting age-specific dietary patterns [11].

The association we observed between added sugars intake and total HEI score was also apparent for the majority of component scores, suggesting that no one particular dietary aspect is driving the association with diet quality. However, a few HEI component scores consistently showed associations in the opposite direction from those observed for the HEI total score. For example, a higher added sugars intake from the combination of soft drinks, fruit drinks and coffee and tea was associated with lower total HEI score (lower overall diet quality) but with higher component scores for total vegetables, total protein foods, fatty acids and saturated fats among children as well as for saturated fats among adults. These findings among children might reflect the co-consumption of these beverages at meals of lean meats and vegetables and perhaps the co-consumption with French fries, particularly among those 9–18 y [28]. In the opposite direction, higher added sugars intake from all foods was associated with higher total HEI score (higher overall diet quality) but with lower scores for those same components (total vegetables, total protein foods, fatty acids and saturated fats among children, and saturated fats among adults). These findings might reflect the displacement of vegetable and protein choices among children consuming more added sugars from all foods and perhaps food sources of added sugars that are also high in saturated fat; whereas among adults, a major source of added sugars is sweet bakery products [11], which are also sources of saturated fats. Our observed discrepancies between the HEI total and component scores suggest that the consumption patterns of different sources of added sugars might also reflect different dietary patterns.

The associations between added sugars intake and diet quality that we reported, although statistically significant, were small in magnitude; the differences in total HEI scores across the quintiles of added sugars were approximately one to two points from the lowest to highest quintile. These findings are similar to those of a systematic review on associations between added sugars intake and diet quality, which found consistent inverse associations across many studies but with small to moderate effect sizes, suggesting limited biological significance [4]. Other researchers have suggested a difference of five to six points in total HEI score as potentially meaningful [29]; however, we do not know if this spread would apply to our analysis, which is based on pooled data from various survey cycles. Furthermore, our calculated HEI scores cannot be interpreted as mean scores for a population group at a particular time, particularly as added sugars intakes have changed over the time period we examined. As such, the practical significance of the differences we observed in total HEI scores is not clear.

Our study provides a unique contribution to the literature, using a large sample size of children and adults from NHANES 2003–2018. New insights emerged regarding differences in the relationship between added sugars intake and diet quality from our comprehensive analysis of added sugars sources at the aggregate and disaggregate levels. Furthermore, by examining children and adults separately, we were able to observe dietary patterns specific to each age group, thereby providing evidence to support age-specific dietary guidance.

As for limitations, our disaggregation of added sugars sources limited our ability to stratify the sample into more age subgroups, or by gender, socioeconomics or race and ethnicity, and thus our analysis does not consider variations in dietary intake according to these factors. Additionally, our findings are specific to the U.S. population and may not generalize to other country contexts due to cultural differences and variations in dietary patterns and food supply. Our analysis is also subject to misreporting errors, which are an inherent part of self-reported dietary intake data; these errors likely affected our estimates of added sugars intake and HEI scores given the evidence of misreporting of various dietary components [29]. However, the significant associations we found between added sugars intake and diet quality are still likely to be true given the fact that misreporting errors can make it more difficult to detect significant associations [30] and given the fact that our findings are consistent with other studies. Lastly, due to the cross-sectional nature of NHANES data, we cannot infer causality between added sugars intake and diet quality.

## 5. Conclusions

In conclusion, our study demonstrates differences in the relationship between added sugars intake and diet quality depending on the added sugars source. Among both children and adults, higher added sugars intake from all beverages, particularly soft drinks, fruit drinks and coffee and tea, was associated with lower overall diet quality; however, higher intake from all foods was associated with higher overall diet quality. Furthermore, children and adults exhibit different dietary patterns related to added sugars intake, and the nature of the relationship between added sugars intake and diet quality depends on the source of added sugars. While the differences in diet quality were statistically significant, they were small in size and thus may be of limited practical significance; however, our results suggest that the consideration of the different roles of various added sugars sources in the diet is warranted when developing dietary guidance.

## Figures and Tables

**Table 1:** Added sugars intake from food and beverage sources among U.S. children and adults (NHANES 2003–2018, *n* = 56,094).

	Children (2–18 y, *n* = 21,000)			Adults (≥19 y, *n* = 35,094)		
	Consumers (%)	Added Sugars (g/Day) Mean ± SE	Added Sugars (% kcal/Day) Mean ± SE	Consumers (%)	Added Sugars (g/Day) Mean ± SE	Added Sugars (% kcal/Day) Mean ± SE
Total diet (all foods and beverages)	99.98	69.85 ± 0.65	14.45 ± 0.09	99.84	68.79 ± 0.67	12.76 ± 0.11
All foods	99.93	38.00 ± 0.34	7.96 ± 0.06	99.58	35.80 ± 0.31	6.76 ± 0.05
All beverages	85.49	31.85 ± 0.56	6.50 ± 0.10	68.65	32.99 ± 0.59	5.99 ± 0.11
Soft drinks, fruit drinks, coffee and tea	75.22	26.43 ± 0.50	5.38 ± 0.10	59.43	28.58 ± 0.60	5.19 ± 0.11
RTEC ^1^	54.49	4.75 ± 0.10	1.04 ± 0.02	30.28	2.37 ± 0.05	0.46 ± 0.01
Flavored milk	26.51	2.58 ± 0.09	0.55 ± 0.02	4.32	0.53 ± 0.03	0.10 ± 0.01
Sweet bakery products	64.22	9.12 ± 0.17	1.85 ± 0.03	55.57	8.97 ± 0.15	1.62 ± 0.02
Snack/meal bars	11.21	0.66 ± 0.03	0.15 ± 0.01	10.07	0.72 ± 0.03	0.15 ± 0.01

^1^ RTEC, ready-to-eat cereals.

**Table 2:** Quintiles of added sugars intake (% kcal) by food and beverage source among U.S. children and adults (NHANES 2003–2018, *n* = 56,094).

	**Children (2–18 y, *n* = 21,000)**				
	Q1 *	**Q2**	**Q3**	**Q4**	**Q5**
Total diet (all foods and beverages)	≤8.65	>8.65 to ≤11.96	>11.96 to ≤15.38	>15.38 to ≤19.73	>19.73
All foods	≤4.27	>4.27 to ≤6.32	>6.32 to ≤8.47	>8.47 to ≤11.29	>11.29
All beverages	≤1.26	>1.26 to ≤3.70	>3.70 to ≤6.59	>6.59 to ≤10.70	>10.70
Soft drinks, fruit drinks, coffee and tea *	0	>0 to ≤2.96	>2.96 to ≤5.62	>5.62 to ≤9.62	>9.62
RTEC ^1,^*	0	>0 to ≤0.84	>0.84 to ≤1.56	<1.56 to ≤2.58	>2.58
Flavored milk *	0	>0 to ≤1.03	>1.03 to ≤1.60	<1.60 to ≤2.56	>2.56
Sweet bakery products *	0	>0 to ≤1.13	>1.13 to ≤2.15	<2.15 to ≤3.85	>3.85
Snack/meal bars *	0	>0 to ≤0.70	>0.70 to ≤1.07	<1.07 to ≤1.63	>1.63
	**Adults (≥19 y, *n* = 35,094)**				
	Q1 *	**Q2**	**Q3**	**Q4**	**Q5**
Total diet (all foods and beverages)	≤5.89	>5.89 to ≤9.41	>9.41 to ≤13.21	>13.21 to ≤18.70	>18.70
All foods	≤2.95	>2.95 to ≤4.99	>4.99 to ≤7.08	>7.08 to ≤9.97	>9.97
All beverages *	0	>0 to ≤3.23	>3.23 to ≤6.59	>6.59 to ≤12.00	>12.00
Soft drinks, fruit drinks, coffee and tea *	0	>0 to ≤3.35	>3.35 to ≤6.58	>6.58 to ≤11.87	>11.87
RTEC ^1,^*	0	>0 to ≤0.57	>0.57 to ≤1.17	>1.17 to ≤2.03	>2.03
Flavored milk *	0	>0 to ≤1.15	>1.15 to ≤1.74	>1.74 to ≤2.80	>2.80
Sweet bakery products *	0	>0 to ≤1.09	>1.09 to ≤2.12	>2.12 to ≤3.80	>3.80
Snack/meal bars *	0	>0 to ≤0.70	>0.70 to ≤1.09	>1.09 to ≤1.79	>1.79

^1^ RTEC, ready-to-eat cereals. * Q1 was set at 0 when the added sugars source was not consumed by >20% of the sample; the remaining sample (those who consumed the source) was divided into four intake groups (Q2, Q3, Q4, Q5).

**Table 3:** Associations between quintiles of added sugars intake and Healthy Eating Index (HEI) 2020 total score by food and beverage source among U.S. children (NHANES 2003–2018, *n* = 21,000).

	Children (2–18 y)						
	Total HEI Score, Mean (SE)					Linear Trend **	
	Q1	Q2	Q3	Q4	Q5	Beta (SE)	*p*-Value
Total diet (all foods and beverages)	52.56 (0.11)	52.25 (0.10)	51.72 (0.10)	51.18 (0.09)	50.73 (0.08)	−0.0978 (0.0057)	<**0.0001**
All foods	51.10 (0.10)	51.67 (0.09)	51.82 (0.09)	51.98 (0.09)	51.88 (0.10)	0.0511 (0.0094)	<**0.0001**
All beverages	53.00 (0.10)	52.40 (0.11)	51.60 (0.09)	51.19 (0.09)	50.25 (0.08)	−0.1477 (0.0070)	<**0.0001**
Soft drinks, fruit drinks, coffee and tea *	52.79 (0.09)	52.54 (0.10)	51.58 (0.10)	51.03 (0.08)	50.17 (0.08)	−0.1559 (0.0077)	<**0.0001**
RTEC ^1,^*	51.12 (0.06)	53.02 (0.12)	52.35 (0.12)	51.95 (0.10)	51.31 (0.10)	−0.0120 (0.0259)	0.6436
Flavored milk *	51.57 (0.06)	51.21 (0.14)	51.77 (0.15)	52.01 (0.14)	53.05 (0.20)	0.3157 (0.0379)	<**0.0001**
Sweet bakery products *	51.23 (0.08)	52.26 (0.10)	52.17 (0.10)	51.78 (0.10)	51.55 (0.11)	0.0029 (0.0161)	0.8573
Snack/meal bars *	51.66 (0.05)	51.16 (0.26)	51.87 (0.25)	52.54 (0.28)	52.03 (0.23)	0.2522 (0.0736)	**0.0008**

Data source: NHANES 2003–2018 based on the average of two days of intake. ^1^ RTEC, ready-to-eat cereals. * Added sugars source was not consumed by >20% of the sample; thus, the HEI score for Q1 is the score for non-consumers. ** From regression analysis, while analyses were conducted for both linear (added sugars intake as a continuous variable) and quintile trends, given the similarity of results for both approaches, only results from the linear trend analysis are presented; bolded *p*-Values are significant at *p* < 0.001.

**Table 4:** Associations between quintiles of added sugars intake and Healthy Eating Index (HEI) 2020 total score by food and beverage source among U.S. adults (NHANES 2003–2018, *n* = 35,094).

	Adults (≥19 y)						
	Total HEI Score, Mean (SE)					Linear Trend **	
	Q1	Q2	Q3	Q4	Q5	Beta (SE)	*p*-Value
Total diet (all foods and beverages)	56.85 (0.09)	57.01 (0.09)	56.73 (0.07)	56.50 (0.09)	56.00 (0.07)	−0.0401 (0.0043)	<**0.0001**
All foods	55.70 (0.08)	56.21 (0.07)	56.51 (0.08)	57.03 (0.07)	57.64 (0.07)	0.1426 (0.0076)	<**0.0001**
All beverages *	57.66 (0.07)	56.85 (0.09)	56.52 (0.08)	55.81 (0.08)	55.36 (0.07)	−0.0991 (0.0051)	<**0.0001**
Soft drinks, fruit drinks, coffee and tea *	57.48 (0.07)	56.64 (0.10)	56.28 (0.08)	55.83 (0.08)	55.37 (0.07)	−0.0939 (0.0051)	<**0.0001**
RTEC ^1,^*	56.51 (0.05)	57.37 (0.12)	56.93 (0.12)	56.68 (0.13)	56.46 (0.13)	−0.0257 (0.0323)	0.4279
Sweet bakery products *	56.26 (0.06)	56.49 (0.09)	56.70 (0.09)	57.03 (0.09)	57.40 (0.09)	0.1519 (0.0140)	<**0.0001**
Snack/meal bars *	56.66 (0.05)	55.66 (0.19)	56.12 (0.18)	56.43 (0.21)	56.73 (0.26)	−0.0092 (0.0661)	0.8894

Data source: NHANES 2003–2018 based on the average of two days of intake. ^1^ RTEC, ready-to-eat cereals. * Added sugars source was not consumed by >20% of the sample; thus, HEI score for Q1 is the score for non-consumers. ** From regression analysis, while analyses were conducted for both linear (added sugars intake as a continuous variable) and quintile trends, given the similarity of results for both approaches, only results from the linear trend analysis are presented; bolded *p*-Values are significant at *p* < 0.001.

## Data Availability

The data used in this study are publicly available at the NHANES website: https://wwwn.cdc.gov/nchs/nhanes/ (accessed on 11 November 2024).

## Supplementary Materials

The following supporting information can be downloaded at https://www.mdpi.com/article/10.3390/nu16244333/s1, Table S1: Associations between quintiles of added sugars intake and Healthy Eating Index (HEI) 2020 total and component scores by food and beverage source among U.S. children (NHANES 2003–2018); Table S2: Associations between quintiles of added sugars intake and Healthy Eating Index (HEI) 2020 total and component scores by food and beverage source among U.S. adults (NHANES 2003–2018).

## Abbreviations

DGA: Dietary Guidelines for Americans; HEI, Healthy Eating Index; RTEC, ready-to-eat cereals; USDA, U.S. Department of Agriculture; WWEIA, What We Eat in America.
