# Supplementary material for: Associations Between Added Sugars Intake from Various Food and Beverage Sources and Diet Quality Among the U.S. Population
Source: Nutrients. 2024 Dec 16;16(24):4333. doi: 10.3390/nu16244333 (PMC11676058; doi:10.3390/nu16244333)
Supplement: Supplementary file 1 [file nutrients-16-04333-s001.zip › nutrients-3337969-supplementary.pdf]

Supplemental Table S1. Associations between quintiles of added sugars intake and Healthy Eating Index (HEI) 2020 total and component scores by food and beverage source among U.S. children (NHANES 2003-2018)

HEI Component	Children (2-18 y, n=21,000)						
	HEI Score, Mean (SE)					Linear Trend**	
	Q1	Q2	Q3	Q4	Q5	Beta (SE)	*P*-value
Total diet (all foods and beverages)							
Total score	52.56 (0.11)	52.25 (0.10)	51.72 (0.10)	51.18 (0.09)	50.73 (0.08)	-0.0978 (0.0057)	**<0.0001**
Total fruits	3.34 (0.02)	3.31 (0.02)	3.23 (0.02)	3.12 (0.02)	3.02 (0.02)	-0.0182 (0.0011)	**<0.0001**
Whole fruits	3.36 (0.02)	3.35 (0.02)	3.27 (0.02)	3.17 (0.02)	3.09 (0.02)	-0.0161 (0.0010)	**<0.0001**
Total vegetables	2.59 (0.01)	2.59 (0.01)	2.58 (0.01)	2.59 (0.01)	2.60 (0.01)	0.0008 (0.0004)	0.0642
Greens + beans	1.72 (0.01)	1.72 (0.01)	1.71 (0.01)	1.70 (0.01)	1.69 (0.01)	-0.0015 (0.0005)	**0.0027**
Whole grains	2.94 (0.01)	2.94 (0.01)	2.90 (0.01)	2.83 (0.01)	2.78 (0.01)	-0.0093 (0.0008)	**<0.0001**
Dairy	8.00 (0.02)	7.96 (0.02)	7.88 (0.02)	7.76 (0.02)	7.64 (0.02)	-0.0203 (0.0013)	**<0.0001**
Total protein foods	4.28 (0.00)	4.28 (0.01)	4.29 (0.00)	4.32 (0.00)	4.33 (0.00)	0.0031 (0.0003)	**<0.0001**
Seafood + plant protein	2.45 (0.01)	2.44 (0.01)	2.42 (0.01)	2.40 (0.01)	2.39 (0.01)	-0.0034 (0.0004)	**<0.0001**
Fatty acids	3.30 (0.01)	3.32 (0.01)	3.34 (0.01)	3.40 (0.01)	3.45 (0.01)	0.0089 (0.0008)	**<0.0001**
Refined grains	4.98 (0.02)	4.89 (0.02)	4.81 (0.02)	4.72 (0.02)	4.70 (0.01)	-0.0138 (0.0010)	**<0.0001**
Sodium	4.34 (0.02)	4.30 (0.02)	4.24 (0.02)	4.13 (0.02)	4.07 (0.02)	-0.0161 (0.0011)	**<0.0001**
Added sugars	6.04 (0.02)	5.96 (0.02)	5.89 (0.02)	5.84 (0.02)	5.76 (0.01)	-0.0140 (0.0010)	**<0.0001**
Saturated fats	5.20 (0.01)	5.18 (0.01)	5.19 (0.01)	5.20 (0.01)	5.23 (0.01)	0.0021 (0.0006)	**0.0018**
All foods							
Total score	51.10 (0.10)	51.67 (0.09)	51.82 (0.09)	51.98 (0.09)	51.88 (0.10)	0.0511 (0.0094)	**<0.0001**
Total fruits	3.05 (0.02)	3.19 (0.02)	3.23 (0.02)	3.28 (0.02)	3.26 (0.02)	0.0141 (0.0017)	**<0.0001**
Whole fruits	3.11 (0.02)	3.23 (0.02)	3.27 (0.02)	3.32 (0.02)	3.31 (0.02)	0.0144 (0.0017)	**<0.0001**
Total vegetables	2.61 (0.01)	2.59 (0.01)	2.59 (0.01)	2.58 (0.01)	2.58 (0.01)	-0.0020 (0.0007)	**0.0058**
Greens + beans	1.69 (0.01)	1.71 (0.01)	1.71 (0.01)	1.71 (0.01)	1.72 (0.01)	0.0014 (0.0009)	0.1326
Whole grains	2.80 (0.01)	2.87 (0.01)	2.88 (0.01)	2.92 (0.01)	2.91 (0.01)	0.0084 (0.0012)	**<0.0001**
Dairy	7.70 (0.02)	7.84 (0.02)	7.88 (0.02)	7.94 (0.02)	7.90 (0.02)	0.0148 (0.0019)	**<0.0001**
Total protein foods	4.34 (0.00)	4.31 (0.00)	4.29 (0.00)	4.28 (0.00)	4.28 (0.01)	-0.0045 (0.0005)	**<0.0001**

Seafood + plant protein	2.39 (0.01)	2.42 (0.01)	2.42 (0.01)	2.43 (0.01)	2.43 (0.01)	0.0026 (0.0007)	**0.0007**
Fatty acids	3.45 (0.01)	3.37 (0.01)	3.35 (0.01)	3.31 (0.01)	3.35 (0.01)	-0.0074 (0.0013)	**<0.0001**
Refined grains	4.77 (0.02)	4.83 (0.02)	4.86 (0.02)	4.83 (0.02)	4.81 (0.02)	0.0005 (0.0015)	0.7298
Sodium	4.08 (0.02)	4.20 (0.02)	4.25 (0.02)	4.28 (0.01)	4.26 (0.02)	0.0120 (0.0018)	**<0.0001**
Added sugars	5.85 (0.01)	5.92 (0.02)	5.90 (0.02)	5.92 (0.02)	5.90 (0.02)	0.0026 (0.0017)	0.1221
Saturated fats	5.25 (0.01)	5.20 (0.01)	5.20 (0.01)	5.18 (0.01)	5.17 (0.01)	-0.0058 (0.0011)	**<0.0001**
All beverages							
Total score	53.00 (0.10)	52.40 (0.11)	51.60 (0.09)	51.19 (0.09)	50.25 (0.08)	-0.1477 (0.0070)	**<0.0001**
Total fruits	3.42 (0.02)	3.36 (0.02)	3.21 (0.02)	3.11 (0.02)	2.92 (0.02)	-0.0298 (0.0012)	**<0.0001**
Whole fruits	3.44 (0.02)	3.39 (0.02)	3.26 (0.02)	3.17 (0.02)	2.98 (0.01)	-0.0273 (0.0012)	**<0.0001**
Total vegetables	2.60 (0.01)	2.58 (0.01)	2.59 (0.01)	2.58 (0.01)	2.61 (0.01)	0.0020 (0.0006)	**0.0010**
Greens + beans	1.74 (0.01)	1.72 (0.01)	1.71 (0.01)	1.69 (0.01)	1.68 (0.01)	-0.0026 (0.0007)	**0.0002**
Whole grains	3.00 (0.01)	2.96 (0.01)	2.87 (0.01)	2.83 (0.01)	2.73 (0.01)	-0.0159 (0.0008)	**<0.0001**
Dairy	8.08 (0.02)	8.03 (0.02)	7.86 (0.02)	7.75 (0.02)	7.54 (0.02)	-0.0327 (0.0014)	**<0.0001**
Total protein foods	4.26 (0.00)	4.26 (0.01)	4.29 (0.00)	4.32 (0.00)	4.36 (0.01)	0.0061 (0.0004)	**<0.0001**
Seafood + plant protein	2.47 (0.01)	2.45 (0.01)	2.41 (0.01)	2.41 (0.01)	2.36 (0.01)	-0.0056 (0.0004)	**<0.0001**
Fatty acids	3.27 (0.01)	3.28 (0.01)	3.35 (0.01)	3.42 (0.01)	3.49 (0.01)	0.0148 (0.0008)	**<0.0001**
Refined grains	5.03 (0.02)	4.89 (0.02)	4.78 (0.01)	4.74 (0.02)	4.66 (0.01)	-0.0175 (0.0012)	**<0.0001**
Sodium	4.41 (0.02)	4.34 (0.02)	4.21 (0.02)	4.14 (0.02)	3.97 (0.01)	-0.0261 (0.0012)	**<0.0001**
Added sugars	6.08 (0.02)	5.97 (0.02)	5.89 (0.02)	5.80 (0.01)	5.73 (0.01)	-0.0187 (0.0012)	**<0.0001**
Saturated fats	5.19 (0.01)	5.16 (0.01)	5.18 (0.01)	5.22 (0.01)	5.24 (0.01)	0.0055 (0.0007)	**<0.0001**
Soft drinks, fruit drinks, coffee and tea*							
Total score	52.79 (0.09)	52.54 (0.10)	51.58 (0.10)	51.03 (0.08)	50.17 (0.08)	-0.1559 (0.0077)	**<0.0001**
Total fruits	3.39 (0.02)	3.38 (0.02)	3.20 (0.02)	3.08 (0.02)	2.90 (0.02)	-0.0314 (0.0013)	**<0.0001**
Whole fruits	3.41 (0.01)	3.41 (0.02)	3.26 (0.02)	3.14 (0.02)	2.96 (0.02)	-0.0287 (0.0013)	**<0.0001**
Total vegetables	2.59 (0.01)	2.58 (0.01)	2.58 (0.01)	2.59 (0.01)	2.61 (0.01)	0.0027 (0.0006)	**<0.0001**
Greens + beans	1.73 (0.01)	1.72 (0.01)	1.70 (0.01)	1.69 (0.01)	1.68 (0.01)	-0.0022 (0.0007)	**0.0026**
Whole grains	2.98 (0.01)	2.96 (0.01)	2.88 (0.01)	2.81 (0.01)	2.71 (0.01)	-0.0173 (0.0008)	**<0.0001**
Dairy	8.06 (0.02)	8.04 (0.02)	7.88 (0.02)	7.70 (0.02)	7.51 (0.02)	-0.0356 (0.0016)	**<0.0001**
Total protein foods	4.27 (0.00)	4.26 (0.00)	4.29 (0.01)	4.32 (0.00)	4.36 (0.01)	0.0063 (0.0004)	**<0.0001**

Seafood + plant protein	2.46 (0.01)	2.45 (0.01)	2.41 (0.01)	2.40 (0.01)	2.35 (0.01)	-0.0058 (0.0005)	**<0.0001**
Fatty acids	3.28 (0.01)	3.28 (0.01)	3.34 (0.01)	3.44 (0.01)	3.51 (0.01)	0.0164 (0.0009)	**<0.0001**
Refined grains	4.99 (0.02)	4.93 (0.02)	4.78 (0.02)	4.72 (0.01)	4.65 (0.01)	-0.0190 (0.0012)	**<0.0001**
Sodium	4.39 (0.02)	4.37 (0.02)	4.21 (0.02)	4.11 (0.02)	3.94 (0.01)	-0.0283 (0.0013)	**<0.0001**
Added sugars	6.05 (0.02)	5.99 (0.02)	5.87 (0.02)	5.80 (0.01)	5.72 (0.01)	-0.0190 (0.0012)	**<0.0001**
Saturated fats	5.18 (0.01)	5.17 (0.01)	5.17 (0.01)	5.22 (0.01)	5.26 (0.01)	0.0060 (0.0007)	**<0.0001**
RTEC*							
Total score	51.12 (0.06)	53.02 (0.12)	52.35 (0.12)	51.95 (0.10)	51.31 (0.10)	-0.0120 (0.0259)	0.6436
Total fruits	3.08 (0.01)	3.45 (0.02)	3.36 (0.02)	3.27 (0.02)	3.15 (0.02)	0.0043 (0.0053)	0.4179
Whole fruits	3.14 (0.01)	3.46 (0.02)	3.38 (0.02)	3.30 (0.02)	3.20 (0.02)	0.0065 (0.0051)	0.2069
Total vegetables	2.61 (0.01)	2.58 (0.01)	2.57 (0.01)	2.57 (0.01)	2.57 (0.01)	-0.0084 (0.0016)	**<0.0001**
Greens + beans	1.71 (0.01)	1.72 (0.01)	1.71 (0.01)	1.71 (0.01)	1.68 (0.01)	-0.0056 (0.0018)	**0.0027**
Whole grains	2.81 (0.01)	3.01 (0.02)	2.93 (0.01)	2.93 (0.02)	2.87 (0.01)	0.0079 (0.0036)	0.0306
Dairy	7.70 (0.01)	8.12 (0.02)	8.02 (0.02)	7.93 (0.02)	7.82 (0.02)	0.0122 (0.0071)	0.0871
Total protein foods	4.32 (0.00)	4.26 (0.01)	4.27 (0.01)	4.28 (0.01)	4.31 (0.01)	-0.0023 (0.0015)	0.1295
Seafood + plant protein	2.40 (0.00)	2.47 (0.01)	2.44 (0.01)	2.43 (0.01)	2.41 (0.01)	0.0001 (0.0020)	0.9505
Fatty acids	3.43 (0.01)	3.24 (0.01)	3.29 (0.02)	3.32 (0.02)	3.37 (0.01)	-0.0089 (0.0043)	0.0384
Refined grains	4.75 (0.01)	5.02 (0.02)	4.92 (0.02)	4.83 (0.02)	4.75 (0.02)	-0.0061 (0.0044)	0.1666
Sodium	4.11 (0.01)	4.44 (0.02)	4.35 (0.02)	4.27 (0.02)	4.17 (0.02)	0.0057 (0.0052)	0.2738
Added sugars	5.84 (0.01)	6.07 (0.02)	5.97 (0.02)	5.92 (0.02)	5.81 (0.02)	-0.0130 (0.0040)	**0.0016**
Saturated fats	5.23 (0.01)	5.16 (0.01)	5.15 (0.01)	5.18 (0.01)	5.20 (0.01)	-0.0044 (0.0032)	0.1822
Flavored milk*							
Total score	51.57 (0.06)	51.21 (0.14)	51.77 (0.15)	52.01 (0.14)	53.05 (0.20)	0.3157 (0.0379)	**<0.0001**
Total fruits	3.17 (0.01)	3.17 (0.03)	3.28 (0.03)	3.34 (0.03)	3.46 (0.04)	0.0697 (0.0069)	**<0.0001**
Whole fruits	3.21 (0.01)	3.24 (0.02)	3.33 (0.03)	3.38 (0.02)	3.47 (0.03)	0.0628 (0.0065)	**<0.0001**
Total vegetables	2.60 (0.00)	2.54 (0.01)	2.55 (0.01)	2.56 (0.01)	2.58 (0.01)	-0.0089 (0.0019)	**<0.0001**
Greens + beans	1.71 (0.00)	1.69 (0.01)	1.68 (0.01)	1.70 (0.01)	1.73 (0.01)	0.0027 (0.0019)	0.1629
Whole grains	2.86 (0.01)	2.88 (0.02)	2.92 (0.02)	2.93 (0.02)	3.01 (0.02)	0.0356 (0.0042)	**<0.0001**
Dairy	7.79 (0.01)	7.87 (0.03)	7.99 (0.03)	8.01 (0.03)	8.14 (0.04)	0.0836 (0.0078)	**<0.0001**
Total protein foods	4.31 (0.00)	4.29 (0.01)	4.27 (0.01)	4.26 (0.01)	4.25 (0.01)	-0.0150 (0.0016)	**<0.0001**

Seafood + plant protein	2.41 (0.00)	2.41 (0.01)	2.42 (0.01)	2.44 (0.01)	2.47 (0.01)	0.0128 (0.0022)	**<0.0001**
Fatty acids	3.39 (0.01)	3.34 (0.02)	3.27 (0.02)	3.28 (0.02)	3.26 (0.02)	-0.0378 (0.0044)	**<0.0001**
Refined grains	4.83 (0.01)	4.68 (0.02)	4.78 (0.03)	4.77 (0.02)	5.01 (0.03)	0.0332 (0.0072)	**<0.0001**
Sodium	4.18 (0.01)	4.17 (0.03)	4.29 (0.02)	4.29 (0.03)	4.46 (0.03)	0.0624 (0.0065)	**<0.0001**
Added sugars	5.90 (0.01)	5.77 (0.02)	5.85 (0.03)	5.90 (0.03)	6.05 (0.03)	0.0314 (0.0065)	**<0.0001**
Saturated fats	5.22 (0.01)	5.17 (0.01)	5.13 (0.02)	5.14 (0.02)	5.16 (0.02)	-0.0167 (0.0035)	**<0.0001**
Sweet bakery products*							
Total score	51.23 (0.08)	52.26 (0.10)	52.17 (0.10)	51.78 (0.10)	51.55 (0.11)	0.0029 (0.0161)	0.8573
Total fruits	3.10 (0.01)	3.31 (0.02)	3.31 (0.02)	3.24 (0.02)	3.20 (0.02)	0.0060 (0.0029)	0.0411
Whole fruits	3.15 (0.01)	3.34 (0.02)	3.35 (0.02)	3.27 (0.02)	3.25 (0.02)	0.0069 (0.0028)	0.0160
Total vegetables	2.60 (0.01)	2.58 (0.01)	2.59 (0.01)	2.58 (0.01)	2.59 (0.01)	-0.0013 (0.0011)	0.2592
Greens + beans	1.70 (0.01)	1.71 (0.01)	1.73 (0.01)	1.71 (0.01)	1.71 (0.01)	0.0008 (0.0013)	0.5489
Whole grains	2.83 (0.01)	2.94 (0.01)	2.91 (0.01)	2.89 (0.01)	2.87 (0.01)	0.0003 (0.0022)	0.8939
Dairy	7.73 (0.02)	7.97 (0.02)	7.95 (0.02)	7.91 (0.02)	7.83 (0.02)	0.0042 (0.0035)	0.2389
Total protein foods	4.32 (0.00)	4.28 (0.01)	4.28 (0.00)	4.29 (0.01)	4.29 (0.01)	-0.0032 (0.0009)	**0.0010**
Seafood + plant protein	2.40 (0.01)	2.44 (0.01)	2.44 (0.01)	2.42 (0.01)	2.41 (0.01)	-0.0004 (0.0012)	0.7533
Fatty acids	3.42 (0.01)	3.31 (0.01)	3.32 (0.01)	3.33 (0.01)	3.37 (0.01)	-0.0029 (0.0021)	0.1586
Refined grains	4.78 (0.01)	4.91 (0.02)	4.87 (0.02)	4.83 (0.02)	4.78 (0.02)	-0.0049 (0.0028)	0.0887
Sodium	4.13 (0.01)	4.32 (0.02)	4.29 (0.02)	4.25 (0.02)	4.20 (0.02)	0.0039 (0.0032)	0.2273
Added sugars	5.85 (0.01)	5.97 (0.02)	5.94 (0.02)	5.90 (0.02)	5.88 (0.02)	-0.0004 (0.0022)	0.8618
Saturated fats	5.23 (0.01)	5.19 (0.01)	5.19 (0.01)	5.17 (0.01)	5.18 (0.01)	-0.0061 (0.0018)	**0.0008**
Snack/meal bars*							
Total score	51.66 (0.05)	51.16 (0.26)	51.87 (0.25)	52.54 (0.28)	52.03 (0.23)	0.2522 (0.0736)	**0.0008**
Total fruits	3.20 (0.01)	3.12 (0.05)	3.23 (0.05)	3.39 (0.05)	3.21 (0.04)	0.0321 (0.0152)	0.0361
Whole fruits	3.24 (0.01)	3.18 (0.05)	3.28 (0.05)	3.41 (0.04)	3.25 (0.04)	0.0311 (0.0130)	0.0179
Total vegetables	2.59 (0.00)	2.61 (0.02)	2.59 (0.02)	2.59 (0.02)	2.65 (0.02)	0.0163 (0.0052)	**0.0022**
Greens + beans	1.70 (0.00)	1.71 (0.02)	1.71 (0.02)	1.74 (0.02)	1.76 (0.02)	0.0187 (0.0050)	**0.0003**
Whole grains	2.87 (0.01)	2.81 (0.03)	2.89 (0.03)	2.98 (0.03)	2.89 (0.03)	0.0201 (0.0094)	0.0341
Dairy	7.85 (0.01)	7.71 (0.06)	7.86 (0.06)	8.03 (0.05)	7.77 (0.06)	0.0071 (0.0179)	0.6916
Total protein foods	4.30 (0.00)	4.30 (0.02)	4.29 (0.02)	4.25 (0.01)	4.30 (0.01)	-0.0068 (0.0038)	0.0756

Seafood + plant protein	2.42 (0.00)	2.43 (0.03)	2.44 (0.02)	2.44 (0.02)	2.45 (0.02)	0.0140 (0.0056)	0.0131
Fatty acids	3.36 (0.01)	3.45 (0.04)	3.36 (0.04)	3.32 (0.04)	3.44 (0.04)	0.0130 (0.0135)	0.3375
Refined grains	4.82 (0.01)	4.66 (0.05)	4.85 (0.04)	4.88 (0.06)	4.89 (0.04)	0.0337 (0.0150)	0.0260
Sodium	4.21 (0.01)	4.12 (0.05)	4.25 (0.05)	4.34 (0.05)	4.22 (0.04)	0.0257 (0.0159)	0.1090
Added sugars	5.89 (0.01)	5.81 (0.04)	5.91 (0.04)	5.98 (0.05)	5.94 (0.04)	0.0244 (0.0153)	0.1121
Saturated fats	5.20 (0.01)	5.25 (0.04)	5.21 (0.03)	5.20 (0.03)	5.26 (0.04)	0.0226 (0.0112)	0.0464

Data source: NHANES 2003-2018 based on the average of two days of intake

*Added sugars source was not consumed by >20% of the sample; thus, HEI score for Q1 is the score for non-consumers

**From regression analysis, while analyses were conducted for both linear (added sugars intake as a continuous variable) and quintile trends, given the similarity of results for both approaches, only results from the linear trend analysis are presented

Supplemental Table S2. Associations between quintiles of added sugars intake and Healthy Eating Index (HEI) 2020 total and component scores by food and beverage source among U.S. adults (NHANES 2003-2018)

HEI Component	Adults (≥19 y, n=35,094)						
	HEI Score, Mean (SE)					Linear Trend**	
	Q1	Q2	Q3	Q4	Q5	Beta (SE)	*P*-value
Total diet (all foods and beverages)							
Total score	56.85 (0.09)	57.01 (0.09)	56.73 (0.07)	56.50 (0.09)	56.00 (0.07)	-0.0401 (0.0043)	**<0.0001**
Total fruits	2.74 (0.01)	2.77 (0.01)	2.74 (0.01)	2.70 (0.01)	2.63 (0.01)	-0.0056 (0.0006)	**<0.0001**
Whole fruits	3.14 (0.01)	3.17 (0.01)	3.13 (0.01)	3.08 (0.02)	3.00 (0.01)	-0.0071 (0.0007)	**<0.0001**
Total vegetables	3.65 (0.01)	3.67 (0.01)	3.64 (0.01)	3.62 (0.01)	3.58 (0.01)	-0.0032 (0.0004)	**<0.0001**
Greens + beans	2.79 (0.01)	2.80 (0.01)	2.78 (0.01)	2.76 (0.01)	2.75 (0.01)	-0.0019 (0.0004)	**<0.0001**
Whole grains	3.00 (0.01)	3.03 (0.02)	2.98 (0.01)	2.93 (0.02)	2.83 (0.01)	-0.0079 (0.0007)	**<0.0001**
Dairy	5.72 (0.01)	5.72 (0.01)	5.71 (0.01)	5.71 (0.01)	5.72 (0.01)	-0.0002 (0.0004)	0.7019
Total protein foods	4.78 (0.00)	4.77 (0.00)	4.77 (0.00)	4.78 (0.00)	4.77 (0.00)	-0.0004 (0.0001)	**<0.0001**
Seafood + plant protein	3.45 (0.01)	3.45 (0.01)	3.43 (0.01)	3.41 (0.01)	3.37 (0.01)	-0.0036 (0.0004)	**<0.0001**
Fatty acids	4.76 (0.01)	4.77 (0.01)	4.76 (0.01)	4.74 (0.01)	4.72 (0.01)	-0.0021 (0.0004)	**<0.0001**
Refined grains	6.50 (0.01)	6.51 (0.01)	6.47 (0.01)	6.46 (0.01)	6.38 (0.01)	-0.0054 (0.0006)	**<0.0001**
Sodium	3.38 (0.01)	3.39 (0.01)	3.39 (0.01)	3.39 (0.01)	3.39 (0.01)	0.0005 (0.0004)	0.2159
Added sugars	7.00 (0.01)	7.01 (0.01)	6.97 (0.01)	6.97 (0.01)	6.88 (0.01)	-0.0052 (0.0006)	**<0.0001**
Saturated fats	5.93 (0.01)	5.94 (0.01)	5.94 (0.01)	5.95 (0.01)	5.98 (0.01)	0.0019 (0.0004)	**<0.0001**
All foods							
Total score	55.70 (0.08)	56.21 (0.07)	56.51 (0.08)	57.03 (0.07)	57.64 (0.07)	0.1426 (0.0076)	**<0.0001**
Total fruits	2.59 (0.01)	2.66 (0.01)	2.70 (0.01)	2.78 (0.01)	2.86 (0.01)	0.0201 (0.0011)	**<0.0001**
Whole fruits	2.95 (0.01)	3.03 (0.01)	3.09 (0.01)	3.18 (0.01)	3.28 (0.01)	0.0245 (0.0013)	**<0.0001**
Total vegetables	3.54 (0.01)	3.59 (0.01)	3.62 (0.01)	3.67 (0.01)	3.73 (0.01)	0.0136 (0.0007)	**<0.0001**
Greens + beans	2.71 (0.01)	2.75 (0.01)	2.77 (0.01)	2.81 (0.01)	2.86 (0.01)	0.0115 (0.0007)	**<0.0001**
Whole grains	2.80 (0.01)	2.89 (0.01)	2.94 (0.01)	3.03 (0.01)	3.12 (0.01)	0.0232 (0.0013)	**<0.0001**
Dairy	5.69 (0.01)	5.71 (0.01)	5.70 (0.01)	5.73 (0.01)	5.75 (0.01)	0.0042 (0.0008)	**<0.0001**
Total protein foods	4.78 (0.00)	4.77 (0.00)	4.78 (0.00)	4.77 (0.00)	4.77 (0.00)	-0.0001 (0.0001)	0.5464

Seafood + plant protein	3.34 (0.01)	3.39 (0.01)	3.41 (0.01)	3.46 (0.01)	3.51 (0.01)	0.0125 (0.0008)	**<0.0001**
Fatty acids	4.70 (0.01)	4.73 (0.01)	4.74 (0.01)	4.77 (0.01)	4.81 (0.01)	0.0080 (0.0008)	**<0.0001**
Refined grains	6.33 (0.01)	6.42 (0.01)	6.46 (0.01)	6.52 (0.01)	6.59 (0.01)	0.0187 (0.0011)	**<0.0001**
Sodium	3.39 (0.01)	3.39 (0.01)	3.39 (0.01)	3.39 (0.01)	3.38 (0.01)	-0.0006 (0.0007)	0.3902
Added sugars	6.90 (0.01)	6.93 (0.01)	6.95 (0.01)	7.00 (0.01)	7.03 (0.01)	0.0100 (0.0010)	**<0.0001**
Saturated fats	5.98 (0.01)	5.95 (0.01)	5.94 (0.01)	5.93 (0.01)	5.94 (0.01)	-0.0029 (0.0008)	**0.0003**
All beverages*							
Total score	57.66 (0.07)	56.85 (0.09)	56.52 (0.08)	55.81 (0.08)	55.36 (0.07)	-0.0991 (0.0051)	**<0.0001**
Total fruits	2.87 (0.01)	2.75 (0.01)	2.70 (0.01)	2.60 (0.01)	2.54 (0.01)	-0.0139 (0.0007)	**<0.0001**
Whole fruits	3.29 (0.01)	3.15 (0.02)	3.09 (0.01)	2.97 (0.01)	2.89 (0.01)	-0.0173 (0.0009)	**<0.0001**
Total vegetables	3.72 (0.01)	3.66 (0.01)	3.63 (0.01)	3.56 (0.01)	3.52 (0.01)	-0.0087 (0.0005)	**<0.0001**
Greens + beans	2.85 (0.01)	2.79 (0.01)	2.77 (0.01)	2.72 (0.01)	2.70 (0.01)	-0.0064 (0.0005)	**<0.0001**
Whole grains	3.14 (0.01)	3.00 (0.02)	2.94 (0.01)	2.82 (0.01)	2.73 (0.01)	-0.0178 (0.0009)	**<0.0001**
Dairy	5.75 (0.01)	5.70 (0.01)	5.71 (0.01)	5.68 (0.01)	5.71 (0.01)	-0.0017 (0.0005)	**0.0003**
Total protein foods	4.78 (0.00)	4.78 (0.00)	4.77 (0.00)	4.77 (0.00)	4.77 (0.00)	-0.0005 (0.0001)	**<0.0001**
Seafood + plant protein	3.51 (0.01)	3.45 (0.01)	3.42 (0.01)	3.35 (0.01)	3.31 (0.01)	-0.0088 (0.0005)	**<0.0001**
Fatty acids	4.80 (0.01)	4.77 (0.01)	4.75 (0.01)	4.71 (0.01)	4.68 (0.01)	-0.0053 (0.0005)	**<0.0001**
Refined grains	6.59 (0.01)	6.50 (0.01)	6.46 (0.01)	6.37 (0.01)	6.29 (0.01)	-0.0131 (0.0007)	**<0.0001**
Sodium	3.38 (0.00)	3.40 (0.01)	3.38 (0.01)	3.39 (0.01)	3.40 (0.01)	0.0008 (0.0004)	0.0597
Added sugars	7.06 (0.01)	6.99 (0.01)	6.95 (0.01)	6.90 (0.01)	6.84 (0.01)	-0.0099 (0.0006)	**<0.0001**
Saturated fats	5.92 (0.01)	5.93 (0.01)	5.95 (0.01)	5.96 (0.01)	6.00 (0.01)	0.0034 (0.0004)	**<0.0001**
Soft drinks, fruit drinks, coffee and tea*							
Total score	57.48 (0.07)	56.64 (0.10)	56.28 (0.08)	55.83 (0.08)	55.37 (0.07)	-0.0939 (0.0051)	**<0.0001**
Total fruits	2.84 (0.01)	2.72 (0.01)	2.66 (0.01)	2.60 (0.01)	2.54 (0.01)	-0.0132 (0.0008)	**<0.0001**
Whole fruits	3.25 (0.01)	3.12 (0.02)	3.04 (0.01)	2.97 (0.01)	2.89 (0.01)	-0.0164 (0.0009)	**<0.0001**
Total vegetables	3.71 (0.01)	3.64 (0.01)	3.60 (0.01)	3.56 (0.01)	3.52 (0.01)	-0.0083 (0.0005)	**<0.0001**
Greens + beans	2.84 (0.01)	2.77 (0.01)	2.76 (0.01)	2.72 (0.01)	2.70 (0.01)	-0.0060 (0.0005)	**<0.0001**
Whole grains	3.11 (0.01)	2.98 (0.02)	2.89 (0.01)	2.82 (0.01)	2.73 (0.01)	-0.0171 (0.0009)	**<0.0001**
Dairy	5.75 (0.01)	5.70 (0.01)	5.69 (0.01)	5.68 (0.01)	5.71 (0.01)	-0.0017 (0.0005)	**0.0006**
Total protein foods	4.78 (0.00)	4.78 (0.00)	4.77 (0.00)	4.77 (0.00)	4.77 (0.00)	-0.0005 (0.0001)	**<0.0001**

Seafood + plant protein	3.49 (0.01)	3.43 (0.01)	3.40 (0.01)	3.35 (0.01)	3.31 (0.01)	-0.0082 (0.0005)	**<0.0001**
Fatty acids	4.79 (0.01)	4.75 (0.01)	4.74 (0.01)	4.71 (0.01)	4.68 (0.01)	-0.0048 (0.0005)	**<0.0001**
Refined grains	6.57 (0.01)	6.47 (0.01)	6.43 (0.01)	6.37 (0.01)	6.29 (0.01)	-0.0125 (0.0007)	**<0.0001**
Sodium	3.38 (0.00)	3.39 (0.01)	3.39 (0.01)	3.39 (0.01)	3.40 (0.01)	0.0008 (0.0004)	0.0542
Added sugars	7.04 (0.01)	6.97 (0.01)	6.94 (0.01)	6.90 (0.01)	6.84 (0.01)	-0.0095 (0.0006)	**<0.0001**
Saturated fats	5.93 (0.01)	5.92 (0.01)	5.96 (0.01)	5.97 (0.01)	6.00 (0.01)	0.0034 (0.0005)	**<0.0001**
RTEC*							
Total score	56.51 (0.05)	57.37 (0.12)	56.93 (0.12)	56.68 (0.13)	56.46 (0.13)	-0.0257 (0.0323)	0.4279
Total fruits	2.70 (0.01)	2.83 (0.02)	2.76 (0.02)	2.75 (0.02)	2.69 (0.02)	-0.0009 (0.0046)	0.8421
Whole fruits	3.09 (0.01)	3.23 (0.02)	3.16 (0.02)	3.13 (0.02)	3.07 (0.02)	-0.0056 (0.0053)	0.2868
Total vegetables	3.62 (0.00)	3.69 (0.01)	3.66 (0.01)	3.63 (0.01)	3.63 (0.01)	-0.0019 (0.0034)	0.5759
Greens + beans	2.77 (0.00)	2.81 (0.01)	2.79 (0.01)	2.77 (0.01)	2.77 (0.01)	-0.0043 (0.0037)	0.2540
Whole grains	2.93 (0.01)	3.12 (0.02)	3.01 (0.02)	2.99 (0.02)	2.92 (0.02)	-0.0036 (0.0051)	0.4758
Dairy	5.71 (0.00)	5.73 (0.01)	5.73 (0.01)	5.73 (0.01)	5.74 (0.01)	0.0086 (0.0030)	0.0051
Total protein foods	4.77 (0.00)	4.78 (0.00)	4.77 (0.00)	4.77 (0.00)	4.77 (0.00)	-0.0011 (0.0007)	0.1229
Seafood + plant protein	3.41 (0.00)	3.50 (0.01)	3.44 (0.01)	3.42 (0.01)	3.41 (0.01)	-0.0030 (0.0031)	0.3271
Fatty acids	4.74 (0.00)	4.79 (0.01)	4.76 (0.01)	4.74 (0.01)	4.76 (0.01)	0.0007 (0.0033)	0.8211
Refined grains	6.45 (0.01)	6.56 (0.02)	6.52 (0.02)	6.46 (0.02)	6.44 (0.02)	-0.0089 (0.0049)	0.0702
Sodium	3.39 (0.00)	3.38 (0.01)	3.39 (0.01)	3.38 (0.01)	3.36 (0.01)	-0.0067 (0.0026)	0.0104
Added sugars	6.96 (0.01)	7.04 (0.02)	7.01 (0.02)	6.97 (0.02)	6.94 (0.02)	-0.0057 (0.0039)	0.1471
Saturated fats	5.95 (0.00)	5.92 (0.01)	5.92 (0.01)	5.95 (0.01)	5.98 (0.01)	0.0067 (0.0036)	0.0637
Sweet bakery products*							
Total score	56.26 (0.06)	56.49 (0.09)	56.70 (0.09)	57.03 (0.09)	57.40 (0.09)	0.1519 (0.0140)	**<0.0001**
Total fruits	2.67 (0.01)	2.70 (0.01)	2.73 (0.01)	2.77 (0.01)	2.83 (0.01)	0.0218 (0.0021)	**<0.0001**
Whole fruits	3.05 (0.01)	3.08 (0.01)	3.12 (0.02)	3.18 (0.02)	3.24 (0.02)	0.0268 (0.0024)	**<0.0001**
Total vegetables	3.60 (0.01)	3.62 (0.01)	3.64 (0.01)	3.66 (0.01)	3.71 (0.01)	0.0137 (0.0012)	**<0.0001**
Greens + beans	2.76 (0.01)	2.76 (0.01)	2.78 (0.01)	2.80 (0.01)	2.83 (0.01)	0.0098 (0.0012)	**<0.0001**
Whole grains	2.89 (0.01)	2.94 (0.02)	2.97 (0.02)	3.03 (0.02)	3.10 (0.02)	0.0276 (0.0026)	**<0.0001**
Dairy	5.72 (0.01)	5.70 (0.01)	5.71 (0.01)	5.71 (0.01)	5.74 (0.01)	0.0019 (0.0015)	0.1931
Total protein foods	4.77 (0.00)	4.78 (0.00)	4.77 (0.00)	4.78 (0.00)	4.77 (0.00)	0.0001 (0.0003)	0.7293

Seafood + plant protein	3.38 (0.01)	3.42 (0.01)	3.43 (0.01)	3.46 (0.01)	3.49 (0.01)	0.0135 (0.0013)	**<0.0001**
Fatty acids	4.73 (0.00)	4.75 (0.01)	4.75 (0.01)	4.77 (0.01)	4.79 (0.01)	0.0088 (0.0014)	**<0.0001**
Refined grains	6.41 (0.01)	6.46 (0.01)	6.48 (0.02)	6.52 (0.01)	6.57 (0.01)	0.0201 (0.0019)	**<0.0001**
Sodium	3.39 (0.00)	3.38 (0.01)	3.39 (0.01)	3.39 (0.01)	3.39 (0.01)	0.0004 (0.0011)	0.7388
Added sugars	6.93 (0.01)	6.96 (0.01)	6.97 (0.01)	7.02 (0.01)	7.03 (0.01)	0.0142 (0.0017)	**<0.0001**
Saturated fats	5.97 (0.01)	5.94 (0.01)	5.94 (0.01)	5.92 (0.01)	5.91 (0.01)	-0.0066 (0.0013)	**<0.0001**
Snack/meal bars*							
Total score	56.66 (0.05)	55.66 (0.19)	56.12 (0.18)	56.43 (0.21)	56.73 (0.26)	-0.0092 (0.0661)	0.8894
Total fruits	2.73 (0.01)	2.55 (0.03)	2.62 (0.03)	2.69 (0.03)	2.72 (0.03)	-0.0093 (0.0086)	0.2778
Whole fruits	3.11 (0.01)	2.92 (0.04)	3.01 (0.03)	3.07 (0.04)	3.12 (0.04)	-0.0061 (0.0097)	0.5298
Total vegetables	3.64 (0.00)	3.54 (0.02)	3.59 (0.02)	3.62 (0.02)	3.64 (0.02)	-0.0001 (0.0063)	0.9899
Greens + beans	2.78 (0.00)	2.71 (0.02)	2.75 (0.02)	2.78 (0.02)	2.82 (0.03)	0.0125 (0.0075)	0.0985
Whole grains	2.97 (0.01)	2.80 (0.03)	2.85 (0.03)	2.90 (0.03)	2.93 (0.04)	-0.0204 (0.0102)	0.0474
Dairy	5.72 (0.00)	5.64 (0.02)	5.69 (0.03)	5.74 (0.03)	5.79 (0.02)	0.0165 (0.0062)	**0.0090**
Total protein foods	4.77 (0.00)	4.78 (0.00)	4.77 (0.01)	4.76 (0.01)	4.76 (0.00)	-0.0027 (0.0012)	0.0236
Seafood + plant protein	3.43 (0.00)	3.36 (0.02)	3.39 (0.02)	3.40 (0.02)	3.41 (0.03)	-0.0055 (0.0062)	0.3784
Fatty acids	4.75 (0.00)	4.70 (0.02)	4.75 (0.02)	4.74 (0.02)	4.76 (0.02)	0.0034 (0.0067)	0.6113
Refined grains	6.47 (0.01)	6.39 (0.03)	6.44 (0.03)	6.41 (0.04)	6.46 (0.04)	-0.0012 (0.0104)	0.9046
Sodium	3.39 (0.00)	3.39 (0.02)	3.38 (0.02)	3.39 (0.02)	3.38 (0.02)	-0.0037 (0.0063)	0.5556
Added sugars	6.97 (0.01)	6.91 (0.03)	6.90 (0.03)	6.93 (0.03)	6.90 (0.04)	-0.0242 (0.0095)	0.0124
Saturated fats	5.94 (0.00)	5.98 (0.02)	5.97 (0.02)	6.00 (0.03)	6.04 (0.02)	0.0317 (0.0061)	**<0.0001**

Data source: NHANES 2003-2018 based on the average of two days of intake

*Added sugars source was not consumed by >20% of the sample; thus, HEI score for Q1 is the score for non-consumers

**From regression analysis, while analyses were conducted for both linear (added sugars intake as a continuous variable) and quintile trends, given the similarity of results for both approaches, only results for the linear trend analysis are presented
